# Could lactate clearance be a marker of mortality in pediatric intensive care unit?

**DOI:** 10.55730/1300-0144.5522

**Published:** 2022-10-09

**Authors:** Özlem SARAÇ SANDAL, Gökhan CEYLAN, Ferhat SARI, Gülhan ATAKUL, Mustafa ÇOLAK, Sevgi TOPAL, Ekin SOYDAN, Utku KARAARSLAN, Hasan AĞIN

**Affiliations:** Department of Pediatric Intensive Care Unit, Dr. Behçet Uz Pediatrics Training and Research Hospital, İzmir, Turkey

**Keywords:** Hyperlactatemia, lactate clearance, intensive care unit, biomarker

## Abstract

**Background/aim:**

Hyperlactatemia is a common finding in critically ill patients and has significant prognostic implications. However, a single lactate measurement has not been correlated to mortality consistently. In this study, we aimed to correlate the clinical efficacy of lactate clearance for the prediction of mortality in pediatric intensive care unit patients.

**Materials and methods:**

This retrospective observational study was performed in the pediatric intensive care unit in patients with lactate level >3 mmol/lt. Initial, 6^th^ h, and 24^th^ h lactate levels were recorded and lactate clearance was calculated using these values (lactate level at admission – level 6 h later × 100/lactate level at admission).

**Results:**

A total of 172 patients were included in the study. Forty-four out of 172 patients died. Median (IQR) lactate (mmol/L) at admission was low in those who survived in comparison to nonsurvivors 4.4 (3.1) vs. 5.75 (7.7) (p = 0.002). Clearance at 6^th^ h was significantly lower in those who died (11.7%) than those who survived (36.7) (p = 0.001).

6^th^ h lactate clearance level <20.7% predicted mortality with a sensitivity of 63.6% and specificity of 69.5% along with a positive predictive value of 41.8 and a negative predictive value of 84.8 (p = 0.004). Both lactate levels and lactate clearance values were significantly predictive factors for mortality (p < 0.05). Only a positive moderate correlation was found between the percentage of PRISM-IV % and 6^th^ h lactate level.

**Conclusion:**

The present study revealed that lactate clearance is a simple and rapid risk-stratification tool holding to be a potential biomarker of managing the treatment efficacy of children in the pediatric intensive care unit.

## 1. Introduction

Hyperlactatemia is a common finding in critically ill patients and has significant prognostic value. The severity and duration of lactic acidosis in critically ill patients correlate with overall oxygen debt, organ dysfunction, and mortality. It is an indicator of inadequate oxygenation or tissue perfusion [1–5]. However, a single lactate measurement has not been correlated to mortality consistently. In current studies, in addition to lactate levels, the importance of lactate clearance (LC) is also emphasized in terms of its relationship with mortality [6,7]. Lactate clearance is the rate of decrease in lactate level after resuscitation is started. This showed further promise in predicting mortality. Various studies have shown that persistent hyperlactatemia beyond 24–48 h was associated with mortality, poor neurological outcome, and organ failure [6,8,9].

A study by Nguyen et al. comparing survivors with nonsurvivors has suggested the role of LC as a marker of mortality prediction in adults. For every 10% increase in LC, the probability of death decreased by approximately 11% [6]. Another study by Nazir et al. showed that 24^th^ h lactate clearance appears superior to 6^th^ h lactate clearance in predicting mortality in septic pediatric patients[7].

Therefore, we investigated clinical efficacy of lactate clearance for the prediction of mortality in pediatric intensive care unit patients.

## 2. Materials and methods

The study was conducted according to the guidelines of the Declaration of Helsinki in 1975 as revised in 2000, and approved by the Cumhuriyet University Ethics Committee (grant number: 2020-02/55). This retrospective observational study was performed in the pediatric intensive care unit of the Department of Pediatrics between January 2015 and December 2019. Written informed consent was obtained from all subjects involved in the study. Patients aged from one month to 18 years and patients with serum lactate level above 3 mmol/L were included in the study [10–12], where patients with metabolic disorders having elevated lactate levels and patients deceased before 28^th^ day of the ICU admission period were excluded.

### 2.1. Methodology

#### Step 1

All children admitted in PICU not meeting any of the exclusion criteria were included in the study after taking written and informed consents.

#### Step 2

- Demographic data including age, gender, diagnosis, admission source, nature of outcome (survival/nonsurvival), and lab parameters were recorded for each patient into a standard medical form.
- A standard Pediatric Risk of Mortality (PRISM) IV, Pediatric Index of Mortality (PIM) III questionnaires, and pediatric sequential organ failure assessment (pSOFA) were completed for the study patients meeting with the inclusion criteria during admission.

#### Step 3

- Initial, 6^th^ h and 24^th^ h lactate levels were recorded and lactate clearance was calculated using these values (lactate level at admission–level 6 h later × 100/lactate level at admission). A positive value determines a decrease or clearance of lactate whereas a negative value determines an increase in lactate.

#### Step 4

- At the same time, as a subgroup patients which were diagnosed with sepsis/septic shock (according to the Surviving Sepsis Campaign (SSC) guidelines as the presence of an infection with signs of organ dysfunction) [13] were analyzed about the relationship between lactate levels, lactate clearance, and mortality.
- In these patients, the distinction between infection and colonization was made according to the prominent signs and symptoms of the patients and the follow-up of the infection criteria [13].

### 2.2. Statistical analysis

The distribution of the variables was performed using Shapiro-Wilks test. Quantitative data were presented as mean and standard deviation where qualitative data as median and interquartile range (IQR-25%–75%) values, and also with numbers and percentage. The analysis of the demographic characteristics was completed by the chi-square test or Fisher’s exact test. The comparison of the laboratory findings was completed by student t-test or Mann-Whitney U test. The relation between lactate and lactate clearance levels, and PRISM-IV, pSOFA and PIM-III scores were conducted by Pearson’s correlation (r) analysis. A univariate logistic regression analysis was used to find the odds ratios of lactate and lactate clearance levels on mortality.

Analysis of the area under the curve (AUC) of the receiver operating characteristic (ROC) curve was performed to evaluate the predictive strength. Cut-off points for initial, 6^th^, and 24^th^ h lactate level and lactate clearance were calculated by the ROC curves at the highest sensitivity and specificity, along with the positive predictive value, negative predictive value, and positive and negative likelihood ratios and odds ratio for the prediction of death.

All data were analyzed using Statistical Package for Social Sciences (SPSS) version 20 program. The significance level was set at p < 0.05.

## 3. Results

### 3.1. Patient characteristics

**Figure 1:**
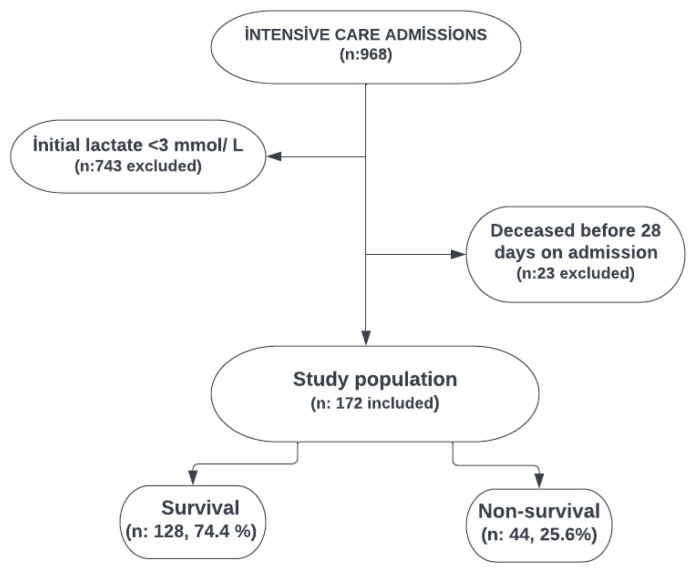
Flow diagram.

The demographic characteristics of the research population are indicated in Table 1. A total of 172 patients were included in the study who fulfilled inclusion criteria as shown in Figure 1. The mean age of the study population (n = 172) was 12 months. Of the 172 children, 89 (51.7%) were females and 44 (26.5%) patients died. The most frequent comorbidity was related to respiratory system, where the intensity of emergency department admission was the highest among others (46.5%). A 25% of the patients was diagnosed with sepsis (Table 1).

### 3.2. Association between lactate kinetics, mortality scores and outcome

Median (IQR) lactate (mmol/L) at admission was low in those who survived in comparison to nonsurvivors 4.4 (3.1) vs. 5.75 (7.7) (p = 0.002). A univariate logistic regression model showed that both lactate levels and lactate clearance values were significantly predictive factors for mortality (Table 2).

Considering the correlation between mortality scores and lactate levels (0, 6, 24 h) and lactate clearance levels (6, 24 h), only a positive moderate correlation was found between the percentage of PRISM-IV% and 6^th^ h lactate level (Table 3).

### 3.3. Serum lactate and clearances cut off of values for predicting mortality

**Figure 2:**
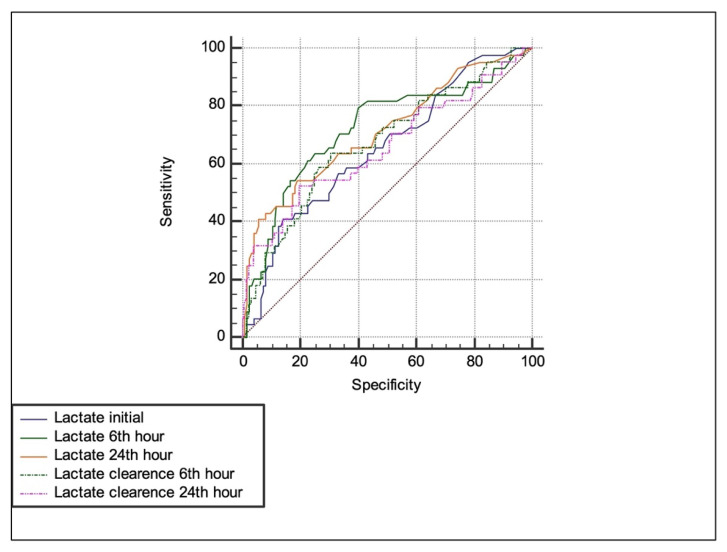
ROC analysis.

Using the area under receiver operating characteristic (ROC) curve, cutoffs for mortality prediction were determined as shown in Figure 2.

An optimal LC cut-off was defined as that LC with the maximum sum of sensitivity plus specificity for predicting inhospital mortality as given in Table 4.

- Serum lactate clearance level at 6 h based on ROC curve demonstrated a clinically acceptable sensitivity and specificity of 63.6% and 69.5% with a cut-off value of 20.7% along with a positive predictive value of 41.8 and a negative predictive value of 84.8 (p = 0.004) which was associated with mortality (Table 4).
- Where the lactate clearance was <20.7% at 6^th^ h, 14 out of 23 died. In those with clearance >20.7% only 30 of 149 died. ROC curve analysis for mortality prediction was 0.67 (p < 0.004).

### 3.4. Subgroup analysis of septic patients

A 25% of the patients were defined as sepsis/septic shock according to the Surviving Sepsis Campaign (SSC) guidelines as the presence of an infection with signs of organ dysfunction. Only three of the patient were without septic shock. Of the total patients, 29 (64.7%) survived and 14 (32.6%) died.

At those the subsequent 6^th^ h and 24^th^ h lactate levels in nonsurvivors were higher than those in survivors (p = 0.004, p = 0.014) (Table 5).

No difference was found between 6^th^ h and 24^th^ h lactate clearance (p = 0.052). It was insufficient data to obtain an acceptable ROC curve analysis and calculate an accurate cut-off level as well in septic patients.

## 4. Discussion

The predictive value of baseline lactate in critically ill patients has been confirmed in several large cohort studies. These studies have demonstrated that serum lactate levels hold a crucial role in risk-stratification in critically ill patients. It is reported during the initial stage of treatment, lactate levels seem to be more closely associated with the outcome compared to frequently measured parameters such as oxygen delivery and oxygen consumption [3,4,8,14].

In a study done by Nguyen et al., serum lactate levels at the time of ED admission were significantly associated with mortality. Mean serum lactate level in nonsurvivors was 8.0 ± 4.7 mmol/L, while lactate level in survivors was 6.1 ± 4.4 mmol/L [6]. This was comparable to our study in which mean serum lactate at 0 h in nonsurvivors was 5.75 ± 2.5 mmol/L and lactate level in survivors was 4.4 ± 2.2 mmol/L. However, unlike this study, in our study, 6^th^ h lactate levels, not the initial lactate level, were correlated with the percentage of PRISM-IV % .

Also Chebl et al. revealed that initial lactate measurement could be a useful tool to clarify critically ill patients regardless of age, or presence of infection [15]. However, earlier studies found controversial findings where Koliski et al. revealed that initial lactate concentration could not be adequate to predict the risk of death and merely 24^th^ h lactate level is able to estimate the survival rate with a sensitivity of 55.6% and a specificity of 97.2% [6,16].

As seen, a single measurement of lactate is a static variable and can only be a risk marker. Previous studies reported that serial lactate measurements are better prognosticators than a single lactate measurement. To make it more clinically useful, lactate clearance was explored during the patients’ stay in the ICU and its relationship with mortality was examined [9,17,18].

A systematic search of the literature reveals scarce findings on the use of lactate measurements for the improvement of the condition of the patient that suggested the requirement of a new tool during medical care [18–22]. In this regard, recent studies have demonstrated the importance of lactate clearance rather than a single measurement [23–28]. Walker et al. conducted a study on children with sepsis and showed that 6^th^ h lactate clearance with a cut-off value of 36% had a sensitivity of 88%, a specificity of 64.1%. Lactate clearance below 36% is closely associated with mortality where survivors had higher lactate clearance values compared to nonsurvivors [22]. Another study has shown a cut-off level of 34.7% with a sensitivity of 87.5% and a specificity of 96.5% in which patients with lower lactate clearance was prone to an increased risk of mortality [19]. The present study has revealed that 6^th^ h lactate clearance level was 20.7% which has a sensitivity of 63.6% and specificity of 69.5% along with a positive predictive value of 41.8 and a negative predictive value of 84.8. We observed higher mortality where the lactate clearance was <20.7% at 6h (p < 0.004) which is similar to the study by Nguyen et al. [6] who observed that clearance <10% in 6^th^ h was associated with higher mortality (p = 0.007). In our study, the cut-off value is higher (20.7%), probably because they studied LC only in cases with a septic patient, while in our study we considered all cases in pediatric intensive care patients.

When septic patients were examined as a subgroup in our study it was found that the subsequent 6^th^ h and 24^th^ h lactate levels in nonsurvivors were higher than those in survivors (p = 0.004, p = 0.014) in septic patients comparable to the study by Nguyen et al. and Kumar et al. [6,17]. However, generalization could not be made due to the small number of patients. Multicenter studies are needed in septic patients.

Besides 6^th^ h lactate level that is moderately correlated with all mortality risk scores, 6^th^ h lactate clearance which also shows a tendency to moderately correlated with the scores in the present study. In relation, a logistic regression model for the prediction of mortality confirmed the previous findings as the odds ratio of 6^th^ h lactate level was 1.144 (CI = 1.060–1.234) and 0.985 (CI = 0.976–0.994) for 6^th^ h lactate clearance. These findings could be accepted as marked evidences of the 6^th^ h lactate clearance is a promising new tool to follow the improvement and recovery of the pediatric patients with pediatric intensive care unit comparable to the study by Kumar et al. [17].

This study is one of the few reports conducted on the importance of lactate clearance in pediatric population and it is more likely to represent a beneficial tool in patients with distinct etiology.

There are some limitations in the present study. First, the initial lactate measurement was performed in the PICU where it could be affected by the previous treatments in the ward before admission to PICU. Second, data were obtained from a single centre which may limit the generalizability of the outcomes. We have provided stratification and cut-off values of lactate clearance which need validation by more multicenter studies with larger samples.

In conclusion, we found 6^th^ h lactate clearance in critically ill patient was associated with mortality prediction. Lactate clearance can probably be used as a simple and rapid risk-stratification screening tool to predict adverse outcomes in all pediatric critical patients. We have provided stratification and cut-off values of lactate clearance which need validation by more studies with larger samples.

## Figures and Tables

**Table 1:** The demographic characteristics of the patients.

	Nonsurvivors (n = 44)	Survivors (n = 128)	Overall (n = 172)	p

**Age (months)** Median(IQR)	12 (59)	12 (81)	12 (80)	0.635^b^

**Gender, n (%)**				0.435^a^
Male	19 (11)	64(37)	83(48)	
Female	25(14)	64(37)	89(51)	

**Diagnosis (n, %)**				

Sepsis/septic shock	14(8.1)	29(16.9)	43(25)	NA

Respiratory	10(5.8)	29(16.9)	39(22.7)	

Cardiovascular	10(5.8)	11(6.4)	21(12.2)	

Neurological	6(3.5)	31(18)	37(21.5)	

Hematological	5(2.9)	18(10.5)	23(13.4)	

Metabolic	6(7.6)	13(3.5)	19(11)	

Intoxication	0(0)	6(3.5)	6(3.5)	

**Admission source (n, %)**				

ED	21(12.2)	59(34.3)	80(46.5)	NA

Ward	13(7.6)	31(18)	44(25.6)	

OT	2(1.2)	1(0.6)	3(1.7)	

AH	8(4.7)	37(21.5)	45(26.2)	

**PICU scores Median (IQR)**				

PRISM-IV	5(9)	14(14)	8(12)	**<0.00** b *

PRISM-IV (%)	2.06(7)	26.85(50)	3.29(22)	**<0.001** b *

pSOFA	4.5(5)	10(6)	5.5(7)	**<0.001** b *

PIM-III	2.4(8.1)	9.35(8.1)	3.25(13.6)	**<0.001** b *

**Lactat Metrics Median (IQR)**				

Lactate _initial_	5.75 (7.7)	4.4(3.1)	4.7(3.7)	**0.002** b *

Lactate _6th h_	5.8(6)	2.8(2.8)	3.25(2.67)	**<0.001** b *

Lactate _24th h_	3.8(9.5)	1.9(1.7)	2.15(2.7)	**<0.001** b *

Clearence _6th h_	11.7(58.51)	36.4(46.76)	32.46(48.86)	**0.001**

Clearence _24th h_	28.38(98.97)	60.43(36.92)	57.9(49.17)	**0.002**

* p<0.05

a Chi-square test.

b Mann-Whitney U test.

NA, not applicable; NLC, no lactate clearance; LC, lactate clearance; ED, emergency department; OT, operating theatre; AH, another hospital; PICU, pediatric intensive care unit; PRISM, Pediatric Risk of Mortality; pSOFA, pediatric sequential organ failure assessment; PIM, Pediatric Index of Mortality.

**Table 2:** Association lactate and mortality.

Variables	OR (95% CI)	p
Lactate _initial_	1.091 (1.017–1.069)	**0.014** *
Lactate _6th h_	1.144 (1.060–1.234)	**0.001** *
Lactate _24th h_	1.211 (1.105–1.326)	**<0.05** *
Clearance _6th h_	0.985 (0.976–0.994)	**0.001** *
Clearance _24th h_	0.985 (0.977–0.992)	**<0.05** *

* p < 0.05

Univariate logistic regression

OR, odds ratio; CI, confidence interval.

**Table 3:** The correlation between lactate levels and mortality scores.

Correlation (r)	PRISM-4	PRISM-4 %	pSOFA	PIM-3
Lactate _initial_	0.463	0.404	0.402	0.408
Lactate _6th h_	0.501	**0.520** *	0.474	0.387
Lactate _24th h_	0.382	0.505	0.483	0.393
Clearence _6th h_	–0.272	–0.334	–0.320	–0.181
Clearence _24th h_	–0.136	–0.290	–0.268	–0.168

* p < 0.05

Pearson correlation analysis.

**Table 4:** Receiver-operating characteristic curve of lactate at 0 h, 6 h, 24 h, lactate clearance 6th h, lactate clearance 24th h rate for predicting mortality.

	Cut-off	AUC	Sensitivity	Specificity	LH+	LH-	PPV	NPV	P
**Lactate ** **_initial_**	>8.1	0.655 (0.562–0.749)	40.9 (26.3–56.8)	85.9 (78.7–91.4)	2.9 (1.7–5.1)	0.7 (0.5–0.9)	50.0 (32.9–67.1)	80.9 (73.3–87.1)	<0.05*
**Lactate ** **_6th h_**	>3.2	0.723 (0.628–0.818)	79.6 (64.7–90.2)	60.2 (51.1–68.7)	2.0 (1.5–2.6)	0.3 (0.2–0.6)	40.7 (30.2–51.8)	89.5 (81.1–95.1)	<0.05*
**Lactate ** **_24th h_**	>3.5	0.714 (0.620–0.808)	54.6 (38.8–69.6)	81.3 (73.4–87.6)	2.9 (1.9–4.6)	0.6 (0.4–0.8)	50.0 (35.2–64.8)	83.9 (76.2–89.9)	<0.05*
**Clearence ** **_6th h_**	≤20.7	0.672 (0.577–0.768)	63.6 (47.8–77.6)	69.5 (60.8–77.4)	2.1 (1.5–2.9)	0.5 (0.3–0.8)	41.8 (29.8–54.5)	84.8 (76.4–91.0)	0.004*
**Clearence ** **_24th h_**	≤28.6	0.656 (0.552–0.759)	52.3 (36.7–67.5)	80.5 (72.5–86.9)	2.7 (1.7–4.2)	0.6 (0.4–0.8)	47.9 (33.3–62.8)	83.1 (75.3–89.2)	0.003*

AUC, area under the curve; LH, likelihood ratio, PPV, positive predictive value, NPV, negative predictive value.

**Table 5:** Lactate kinetics in septic patients.

	Survivors median(IQR)	Nonsurvivors median (IQR)	p
**Lactate ** **_initial_**	5.4 (7.1)	8.6 (7.3)	0.053
**Lactate ** **_6th h_**	4.3 (4.9)	7.4 (8.6)	**0.004** *
**Lactate ** **_24th h_**	2.4 (2.5)	7.2 (14.7)	**0.014** *
**Clearance ** **_6th h_**	32.5 (45)	5.43 (49)	0.052
**Clearance ** **_24th h_**	57.4 (36)	−1.95 (119.3)	0.05

* Mann Whitney U test, p < 0.05
